# Longitudinal Insights into the Mental Health of Healthcare Workers: Emotional Shifts During Two Years of the COVID-19 Crisis

**DOI:** 10.3390/brainsci14111155

**Published:** 2024-11-19

**Authors:** Maia Stanisławska-Kubiak, Grażyna Teusz, Michał Ziarko, Ewa Mojs

**Affiliations:** 1 Department of Clinical Psychology, Poznan University of Medical Sciences, 60-812 Poznan, Poland; ewamojs@ump.edu.pl; 2 Faculty of Educational Studies, Adam Mickiewicz University, 60-568 Poznan, Poland; jasmin8@amu.edu.pl; 3 Faculty of Psychology and Cognitive Sciences (FPCS AMU), Adam Mickiewicz University, 60-568 Poznan, Poland; michal.ziarko@amu.edu.pl

**Keywords:** emotional impact, stress, healthcare professionals, mental health, coping strategies

## Abstract

Objective: Numerous studies have highlighted the prevalence of mental health disorders among healthcare professionals during the COVID-19 pandemic, with varying indications of emotional strain. This study compares the psychological functioning of healthcare workers at the onset of the pandemic and two years later, offering a comprehensive assessment of their emotional and mental health status in the evolving context of COVID-19. Methods: This longitudinal analysis examined the relationship between stress, emotional processing, and their positive/negative impacts on medical personnel working in Polish hospitals and outpatient clinics in 2020 (*n* = 285) and 2022 (*n* = 252). The study employed the Toronto Alexithymia Scale-20 (TAS-20), Cohen’s Perceived Stress Scale (PSS-10), Mini-COPE, Acceptance of Illness Scale (AIS), Emotional Processing Scale (EPS), STAI, and PANAS to assess psychological responses and coping mechanisms. Results: Findings revealed a significant increase in denial, substance use, self-blame, negative mood, and impaired psychological and somatic functioning, alongside heightened symptoms of depression and anxiety. Conversely, a marked decrease in planning, positive reinterpretation, acceptance, religious coping, and seeking social support (both emotional and instrumental) was observed over the two-year period. Conclusion: The prolonged nature of the COVID-19 pandemic has profoundly affected the psychological resilience of healthcare professionals, eroding critical emotional resources necessary for maintaining interpersonal relationships and mental well-being. These results underscore the need for targeted interventions to support the mental health of medical staff in the ongoing crisis.

## 1. Introduction

The Coronavirus 2019 (COVID-19) disease has created a global situation and is responsible for posing a number of medical, ethical, social, and organizational challenges [1]. The rapid spread of the disease in late 2019 and early 2020 took many healthcare systems by surprise. In light of the pandemic, healthcare staff have faced many difficulties that pose a risk to their health, well-being, and the ability to do their jobs [2]. Research has consistently shown that increased levels of work-related stress associated with the pandemic can adversely affect the occupational health and well-being of medical staff. According to meta-analyses, the COVID-19 pandemic is having a significant impact on the mental health of health professionals. The overall prevalence of insomnia, stress, anxiety, and depression among healthcare staff is high [3,4]. However, stress is not a simple construct. It is multi-faceted and complex. Various physiological, psychological, social, and emotional factors can affect stress. Therefore, the reactions of healthcare staff to stress caused by the pandemic need to be considered carefully [5,6,7]. Exposure to COVID-19 patients has been indicated as a risk factor, and several other variables (fatigue, fear, and helplessness resulting from a new situation not previously experienced) have been identified to explain the reported symptoms of depression, anxiety, and insomnia [8]. A number of different situations can be a source of anxiety: becoming infected and also infecting one’s loved ones [9,10,11], confrontation with high rates of disease transmission and mortality [2,9], the unknown in relation to the disease [9], extremely long working hours [10], experiencing many sudden patient deaths, and informing families of the deaths of patients who are unable to say goodbye to them [10].

Healthcare professionals are obliged to provide support to patients [2]. Pursuant to medical ethics, healthcare professionals around the world were obliged to save the lives of others during a pandemic while putting their own lives and health at risk [12]. Heath et al. also demonstrated that healthcare professionals whose job responsibilities interfere with their home life are more likely to experience burnout, leading to high stress when providing patient care [13]. Extended shifts and disrupted biological rhythms during quarantine often led to insomnia [14]. Worry and stress evolved into fear of COVID-19 patients and of healthcare staff who come into contact with them, contributing to greater isolation and a lack of support [15]. Researchers are aware that the process of experiencing stress at the onset of the pandemic has undoubtedly changed the present psychological functioning of health professionals and that the anxiety and stress experienced in the face of the pandemic is a process, which may prevent the emotional state of health professionals returning to its pre-pandemic state.

In the face of the COVID-19 pandemic, which has significantly burdened healthcare workers, it is crucial to understand how occupational stress affects emotional processing and coping mechanisms in medical professions. A foundational theoretical framework for examining the relationship between stress and emotional processing is the transactional model of stress and coping by Lazarus and Folkman (1984). This model defines stress as a transaction between environmental demands and individual resources, where stress arises when these demands exceed one’s adaptive capacities [16]. In healthcare settings, factors such as increased workload, exposure to infected patients, and risk of personal infection serve as powerful stressors that may influence healthcare professionals’ ability to manage emotions effectively.

The prolonged nature of the pandemic underscores how chronic stress and the depletion of psychological resources can weaken coping mechanisms and promote less adaptive emotional processing strategies. Endler and Parker’s (1990) theory of adaptive coping provides further insight, explaining that individuals facing long-term stress are often inclined toward coping strategies that require fewer cognitive and emotional resources. Under crisis conditions, such as a pandemic, these resources may become depleted, leading healthcare workers to adopt avoidance-based or short-term coping strategies. While these may ensure immediate functionality, they are generally less effective for long-term emotional well-being [17,18,19,20].

This study assumes that emotional processing and anxiety coping mechanisms will vary among healthcare workers depending on the pandemic stage and duration of occupational stress exposure. Such an approach allows us to better understand how complex adaptive mechanisms influence mental health and the capacity to manage emotions in a sustained crisis situation.

Hence, research on methods for healthcare staff to cope with excessive strain is incredibly important. The said research focuses on emotional processing, which is related to medical personnel’s ability to manage emotions and cope with a long-lasting crisis.

Given the crucial importance of a better understanding of the psychological responses of staff to the prevailing situation in the healthcare system, factors such as the relationship between stress, the way emotions are processed, and their negative/positive impact on medical staff working in Poland at the start of the pandemic (2020) and two years after (2022) were analyzed within the scope of this study. This study attempts to determine how the duration of the pandemic changes emotional parameters among medical staff. We hypothesized that the events associated with the COVID-19 pandemic have an impact on the way people manage their emotions and cope with anxiety.

This study is one of the first in Poland to adopt a long-term perspective on the mental health of healthcare professionals during the pandemic. By examining both positive and negative coping strategies over two stages of the pandemic, this research offers a comprehensive view of adaptive mechanisms and their long-term implications for mental well-being. Additionally, the well-being of healthcare professionals directly impacts patient care quality, as professionals experiencing high levels of stress and emotional exhaustion may struggle to maintain optimal patient outcomes.

## 2. Materials and Methods

### 2.1. Participants and Procedure

This longitudinal study was conducted across two periods: during the initial wave of the COVID-19 pandemic (March–June 2020) and two years later (March–June 2022). In 2020, a sample of 285 healthcare professionals—including doctors, nurses, and paramedics—was recruited from large hospitals and outpatient clinics located in the Greater Poland and Lesser Poland regions. Participants were initially contacted through institutional emails, and those who consented were provided with a link to an online survey. In 2022, these same participants were invited to complete a follow-up survey, and 252 healthcare professionals participated.

This longitudinal design offers key advantages. First, by repeating measurements over time, we could observe potential shifts in the emotional states of healthcare workers, enabling us to understand the longer-term impacts of the pandemic. Second, studying the same group in both waves helps minimize confounding variables, as individual differences remain constant, allowing us to attribute observed changes more directly to pandemic-related stressors.

A non-random sampling method was applied, focusing on healthcare workers directly involved in patient care during the pandemic. All participants provided informed consent by signing a consent form prior to their involvement in both study phases. To address common method bias, clear inclusion and exclusion criteria were used; only healthcare professionals who voluntarily provided their contact information in the first phase were re-contacted for the follow-up study.

For confidentiality and ethical considerations, we did not collect any personally identifiable data, including participants’ sex, age, years of practice, or COVID-19 infection history, to maintain complete anonymity throughout the study.

### 2.2. Measures

Statistical Analysis: We conducted a normality check using the Kolmogorov–Smirnov test, which revealed that some variables did not meet the assumptions of normality. As a result, non-parametric tests were employed where appropriate. Specifically, we used the Mann–Whitney U test and Spearman’s rank correlation coefficient for non-normally distributed variables, ensuring robust analysis without violating statistical assumptions. This approach allowed us to accurately capture and interpret differences and associations within the data, avoiding biases that could result from parametric analysis of non-normally distributed data.

Ethical Considerations: Informed consent was obtained from all participants, who were assured of their anonymity and the confidentiality of their responses. They were also informed of their right to withdraw from the study at any time without providing a reason. Ethical approval was granted by the University Bioethics Committee.

## 3. Results

The results of the Student’s *t*-test for independent samples (Table 1) showed a significant increase in healthcare staff’s scores between the measure at the beginning of the pandemic and after two years for the following variables. Tests for stress emotional state analysis show a significant increase in negative mood; mental and somatic health disorders; anxiety; dysfunction; symptoms of depression; anxiety, examined both as a state and as a trait; emotion regulation-refinement; and emotion regulation-suppression.

The results also showed a significant decrease (Table 2) in scores between the beginning of the pandemic and two years later for the following variables: planning, positive reinterpretation, acceptance, turning to religion, seeking social support for emotional reasons, seeking social support for instrumental reasons, and positive mood.

The results showed no significant changes (Table 3) for the following variables: acceptance of illness, active coping, venting, sense of humor, and behavioral disengagement.

## 4. Discussion

Research has shown that the COVID-19 pandemic can have a wide-ranging impact on psychological well-being [21]. Mental health in the general population was found to deteriorate during the pandemic compared to the pre-pandemic period. The results of this study are in line with research on the emotional impact of the coronavirus pandemic on healthcare workers [22,23]. The fact that health services were subject to excessive strain is indisputable. A study on a large group of health professionals from 14 countries used the Kessler Psychological Distress Scale (K-10), the Fear of COVID-19 Scale (FCV-19S), and the Brief Resilient Coping Scale (BRCS) to assess psychological distress, fear, and coping, respectively, of health professionals in the context of a pandemic. Moderate to very high psychological distress was observed for 67% of health professionals. This stress is not uniform, and research should take into account individual factors [1]. Perceived anxiety associated with changing jobs, co-morbidities other than mental health problems, and the perceived state of one’s own mental health were significantly correlated with mental distress, fear, and coping in this study. It is therefore very important to recognize the individual needs of healthcare professionals and to take into account the factors that can significantly affect their psychological well-being during such critical periods.

Consequently, the stress of having to work during an epidemiological emergency led to a deterioration of mental health and triggered maladaptive coping strategies. Habitual use of non-adaptive strategies can provide short-term relief by reducing the negative effects of stressful transactions and facilitating mobilization or simply working at sufficient productivity levels. However, in the long term, this can lead to deterioration in health. In a crisis situation, many personal resources are depleted, and for this reason, an individual may be less inclined to use coping strategies that require additional cognitive or emotional resources. Studies emphasize that prolonged exposure to pandemic-related stressors can lead to cumulative health effects, highlighting the need for ongoing psychological support for healthcare workers [24,25].

This research shows that the emotional response to stress caused by the pandemic can be determined by stress, which can determine the strategies activated in the face of a crisis. Research also points to the role of emotional processing, which is linked to the ability to cope with particularly difficult events [26]. The problem of significantly reduced performance in the emotion management skills of helpers is analyzed from many perspectives, but the pandemic crisis undoubtedly reveals more profoundly the weaknesses of the system in place.

This study captures the dynamic mental health impact of the COVID-19 pandemic on healthcare workers across two waves, providing a longitudinal view of changes in psychological well-being. Our findings align with international research demonstrating that healthcare workers have faced an accumulation of mental health challenges as the pandemic continued, with many experiencing adverse psychological effects such as heightened stress and burnout [27,28]. In our study, we observed significant differences in emotional well-being, stress, and coping strategies between the first wave, during the acute crisis, and the second wave, two years later.

Wave One Findings: The initial wave (2020) highlighted an acute rise in stress levels among healthcare workers, a phenomenon also noted in similar studies [29]. During this period, factors such as increased workload, the fear of infection, and emotional exhaustion were especially pronounced. These findings are consistent with research conducted in Poland, where medical personnel reported significant concerns regarding personal safety and emotional burnout [30]. Our study supports the hypothesis that intense exposure to crisis conditions can trigger high levels of distress, often resulting in maladaptive coping strategies that provide immediate, albeit temporary, relief from stress [31].

Wave Two Findings: Two years into the pandemic, we found a slight decrease in reported stress levels but noted that healthcare workers continued to experience considerable psychological strain. This prolonged exposure to pandemic-related stressors, as observed in similar longitudinal studies, appears to lead to cumulative psychological wear and tear, even as individuals attempt to adapt to a “new normal” [28]. While some participants demonstrated resilience, others reported persistent symptoms of anxiety and emotional exhaustion, a pattern of heterogeneous mental health responses documented in Finland [31].

Our findings suggest that although some healthcare workers developed adaptive coping mechanisms over time, others struggled with the prolonged demands of high-stress work environments, which ultimately impacted their emotional processing and ability to manage stress constructively. Previous studies indicate that prolonged exposure to pandemic-related stressors without adequate support can lead to burnout and decreased occupational well-being, underscoring the need for systematic mental health support [28].

Implications for Support Interventions: Our research highlights the critical need for tailored mental health support and the development of resilience-building programs for healthcare workers, particularly during prolonged crisis situations. Training in emotional regulation and adaptive coping could help mitigate the adverse psychological effects seen in these professionals. Implementing these strategies may improve both personal well-being and professional efficacy, enabling healthcare workers to manage stress more sustainably.

Limitations and Future Research: Although this study provides valuable insights, some limitations should be noted. First, while the study offers a longitudinal perspective, the sample was recruited through a non-random sampling method, which may introduce bias. Additionally, due to the anonymous nature of the survey, we could not track individual health changes over time.

## 5. Conclusions

In conclusion, this research reinforces the critical role of structured mental health interventions for healthcare professionals and emphasizes the importance of addressing both immediate and long-term psychological needs. By examining the changes in mental health across two time points, our findings contribute to a deeper understanding of the evolving mental health landscape among healthcare workers throughout the COVID-19 pandemic.

Pandemic-Related Psychological Decline: The findings indicate that prolonged exposure to the COVID-19 pandemic has exacerbated a wide range of psychological symptoms among healthcare professionals. After two years, there is evidence of a marked decline in psychological coping skills. The ability to care for others did not correlate with self-care capacity, suggesting a disconnect between professional caregiving and personal mental well-being.

Insufficient Support Systems: The study highlights a critical lack of psychological support for medical staff during the pandemic. While significant resources were allocated to mitigating the biological risks of COVID-19, stress-related challenges were underestimated, and systemic interventions to alleviate mental health burdens were largely absent.

Implications for Healthcare Management: The factors identified in this study emphasize the need for a more comprehensive approach to managing healthcare workers’ well-being. This should include psychological support at both the training and professional stages, particularly since these individuals are at the frontline of managing crises and dealing with highly stressed and vulnerable patients.

Long-Term Psychological Effects: Even two years after the pandemic’s onset, healthcare workers continued to exhibit mild to moderate adverse psychological symptoms. This study provides a snapshot of the long-term mental health effects of the pandemic on healthcare professionals, highlighting the need for sustained mental health support and intervention to prevent chronic psychological damage.

## Figures and Tables

**Table 1:** Variables for which a significant increase was determined—Student’s *t*-test for independent samples.

Variable	Measure I (*n* = 285)		Measure II (*n* = 252)		*t*	*p*
	M	SD	M	SD		
**Emotional processing**	89.67	44.48	185.38	12.80	−32.96 **	<0.001
**Suppression**	19.18	10.40	37.38	4.33	−25.84 **	<0.001
**No processing**	19.8	11.84	41.97	2.71	−29.20 **	<0.001
**No regulating**	16.03	9.83	34.94	5.18	−27.34 **	<0.001
**Avoidance**	20.58	9.50	37.64	4.26	−26.26 **	<0.001
**Scarce experience**	14.18	10.01	33.43	3.93	−28.61 **	<0.001
**Engagement in other actives**	3.71	1.57	4.17	1.18	−3.77 **	<0.001
**Denial**	1.44	1.52	2.13	0.99	−6.20 **	<0.001
**Use of psychoactive substances**	1.21	1.56	3.64	1.55	−17.98 **	<0.001
**Self-blame**	2.84	1.61	3.90	1.61	−7.56 **	<0.001
**Perceived stress**	19.82	6.91	31.23	3.29	−23.88 **	<0.001
**Negative mood**	25.08	8.62	29.22	3.77	−7.04 **	<0.001
**Mental health disorders**	6.11	6.06	8.20	6.20	−13.93 **	<0.001
**Somatic disorders**	2.00	1.99	3.50	2.32	−11.27 **	<0.001
**Anxiety**	2.36	2.48	3.00	2.11	−9.23 **	<0.001
**Social dysfunction**	1.17	1.77	2.12	1.90	−10.96 **	<0.001
**Symptoms of depression**	0.76	1.41	1.50	1.56	−8.26 **	<0.001
**Phobia: state**	43.73	11.56	54.11	4.69	−13.16 **	<0.001
**Phobia: trait**	42.81	9.48	52.57	4.60	−14.62 **	<0.001
**Emotion regulation-Redefined**	14.46	5.26	20.80	2.33	−17.64 **	<0.001
**Emotion regulation-Suppression**	27.81	7.95	19.09	2.55	−16.67 **	<0.001

** *p* < 0.01; Source: author’s own work.

**Table 2:** Variables for which a significant decrease was determined—Student’s *t*-test for independent samples.

Variable	Measure I (*n* = 285)		Measure II (*n* = 252)		*t*	*p*
	M	SD	M	SD		
**Planning**	4.46	1.29	3.48	1.36	8.57 **	<0.001
**Positive reinterpretation**	3.84	1.45	2.24	1.37	13.07 **	<0.001
**Acceptance**	4.09	1.30	2.19	1.22	17.33 **	<0.001
**Turning to religion**	2.17	2.02	1.24	1.04	6.58 **	<0.001
**Seeking social support for emotional reasons**	3.90	1.58	2.86	1.12	8.68 **	<0.001
**Seeking social support for instrumental reasons**	3.75	1.53	2.32	1.73	10.12 **	<0.001
**Positive mood**	32.07	6.97	29.22	3.77	9.01 **	<0.001

** *p* < 0.01; Source: author’s own work.

**Table 3:** Variables for which no significant changes were determined—Student’s *t*-test for independent samples.

Variable	Measure I (*n* = 285)		Measure II (*n* = 252)		*t*	*p*
	M	SD	M	SD		
**Acceptance of illness**	30.23	8.40	30.59	3.75	−0.62	0.538
**Active coping**	4.53	1.32	4.54	0.76	−0.17	0.866
**Venting**	2.92	1.29				
**Sense of humor**	1.93	1.28	1.88	1.56	0.39	0.697
**Behavioral disengagement**	1.41	1.38	1.52	1.24	−1.03	0.304

Source: author’s own work.

## Data Availability

Due to the possibility of inappropriate data disclosure. We want for our sensitive data to be only accessible to approved parties.
